# Assessing the practicality of using freely available AI-based GPT tools for coach learning and athlete development

**DOI:** 10.3389/fspor.2025.1627685

**Published:** 2025-07-29

**Authors:** Katherine A. O'Brien, Sarah Prentice

**Affiliations:** School of Exercise and Nutrition Sciences, Queensland University of Technology, Brisbane, QLD, Australia

**Keywords:** GPT technology, artificial intelligence, coach learning, athlete development, ChatGPT, DeepSeek, conversational analysis, sport officiating

## Abstract

This study represents one of the initial efforts to analyse a coach-athlete conversational dataset using freely available GPT tools and a pre-determined, context-specific, prompt-based analyses framework (i.e., R^2^-PIASS). One dialogue dataset was selected by means of two different freely available AI-based GPT tools: ChatGPT v4 and DeepSeek v3. The results illustrated that both ChatGPT v4 and DeepSeek v3 models could extract quantitative and qualitative conversational information from the source material using simple R^2^-PIASS prompt specifiers. Implications for how coaches can use this technology to support their own learning, practice designs, and performance analyses were the efficiencies both platforms provided in relation to cost, usability, accessibility and convenience. Despite the strengths, there were also associated risks and pitfalls when using this process such as the strength and robustness of the applicable statistical outcomes and tensions between keeping the input data within the context and ensuring that the context did not breach privacy issues. Further investigations that engage GPT platforms for coach-athlete dialogue analysis are therefore required to ascertain the true relevance and potential of using this type of technology to enhance coach learning and athlete development.

## 1 Introduction

For decades, sports coaching has used technology such as video feedback (VF) to enable observations of performance multiple times. Advanced technologies in wearable sensors to monitor and evaluate physiological biomarkers combined with the use of computer-assisted analysis have also provided sport coaches with cutting-edge insights and the quantification of performance data without direct proximity to athlete practice and performance sessions (1–3). Moreover, recent developments in Artificial Intelligence (AI) technologies now allow sport coaches the chance to review key movement moments alongside quantitative data in real time, providing much faster insights into their athletes' performance results (4). However, while athlete monitoring tools and analysis software are widely seen as standard for shaping athlete development processes, understanding the degree to which the automated collection of data using AI-based algorithms and associated tools can support sport coaches with their professional practice and own learning is not well understood (4, 5). Indeed, few studies have been conducted to determine whether sport coaches are willing to integrate AI-based Generative Pretrained Transformer (GPT) tools into activities that support their practice processes, formal and informal learning, developmental experiences, and self-analyses of their own work. Yet, if we accept that a consistent factor in athlete and team success is the contribution of the coach, positioning how advanced AI-based GPT tools such as Microsoft Copilot, Google Bard, Claude or ChatGPT can support and shape coaching knowledge and processes related to practice design is important (6–8).

The purpose of this project is aligned with assessing the functionality of freely available AI-based GPT tools such as DeepSeek and ChatGPT for sport coach development. Development, in this case, represents the process of a person (i.e., sport officiating coach) seeking to deliberately become a better version of themselves through the incorporation of a digital technology that identifies and analyses human-like language (8, 9). Leveraging how smart language tools can assist with identifying and signposting processes related to human language analysis, such as the diagnosis and delivery of performance feedback, is valuable for understanding whether AI-based GPT tools can support sport coaches with their own learning and athlete development. Moreover, we ask, can ChatGPT, Copilot, Bard and other freely available AI-assisted GPT applications become part of a sport coaches toolkit to be embedded in, replace and/or augment many of the tasks related to the diagnosis and delivery of performance feedback connected to coach-athlete dialogue. For example, data platforms powered by AI analyses and conversational systems based on Large Language Models (LLMs) such as ChatGPT potentially offer online, scalable systems for delivering tailored team- and athlete-based performance feedback (6, 10, 11). Beyond athletes, AI-based GPT tools also hold promise for transforming coaching practices and subsequent learning in areas related to quantifying coach-athlete spoken parameters. General AI platforms that utilise Natural Language Processing (NLP) for instance offer the capacity to statistically analyze language structures, allowing sport coaches to examine the complete meaning of text or speech, including intent and emotions (12). Thus, while the concept of applying AI-based GPT tools to support coach learning and performance analyses might be a fairly recent phenomenon, AI-based GPT tools that act as language interpreters within LLMs have been in existence for several years [see (13)], suggesting that these applications and digital tools might provide an alternative pathway for advancing sport coach learning and athlete development in the conversational analysis field (11, 14).

The aim of this paper is to explore whether freely available AI-based GPT tools can be used to tag, configure, and analyze audio data for the purpose of completing a conversational-based performance assessment. Specifically, conversations between sport referees and players on-field will provide the audio data. Like coach-athlete conversations, referee-player dialogue involves not only the exchange of information but also complex relational and interactive processes that involve negotiated meanings and the building of relationships. To offer a systematic and transparent approach to evaluating whether an AI-based GPT tool can effectively signpost conversational performance parameters, we previously developed and published a novel, prompt-based conversational agent termed the Rugby–Referee–Players' Interaction Assessment Scoring System (R^2^-PIASS) for evaluating referee-player dialogue (O'Brien et al., 2025 under *review*). The framework was initially developed from empirical research on the role of the referee in youth sport and teacher-student interactions [see (15)], and then modified to create a sport specific tool for supporting rugby union referee match coaches with completing referee performance assessments. Thus, while the R^2^-PIASS was designed for examining the network of speech acts that contribute to rugby referees transferring and responding to information about events taking place on field or having taken place (16, 17); (O'Brien et al., 2025), its fundamental design was underpinned by a gap in the research; that is, understanding whether tailored GPT prompts can be leveraged to support sport coaches with refining their coach-athlete feedback related to athlete performance and evaluations of their own practice processes. As such, this study deliberately sought to assess the quality of referee (as the coach)-player conversations by incorporating the R^2^-PIASS parameters as input prompts into freely available AI-based GPT tools. The objective is to evaluate the GPT's ability to structure its responses, essentially using the parameters to act as a customised blueprint for guiding the feedback outputs and practice process suggestions related to coach-player dialogue measures.

## 2 Ethical considerations

This study was approved by the Human Research Ethics Committee of The Queensland University of Technology [QUT] (Ethics: #7278). Informed consent was obtained from all participants before recording their games using an Insta 360-degree video camera. Participants did not receive any monetary compensation for being part of the study and the authors report there are no competing interests to declare. No identifiable or sensitive player information was uploaded into the freely available AI-based GPT tools.

## 3 Materials and methods

The Design Science Research (DSR) paradigm framed the underlying assumptions, beliefs, and approaches used to explore and analyze the conversational data. While DSR is primarily used in the Information Systems (IS) space, it also underscores how to address real-world problems. For example, rather than simply observing and theoretically explaining existing phenomena, the paradigm aims to understand and validate subjective meanings through information or collected data within specific contexts such as coach learning, performance settings, or athlete development (18). This meant that a strong emphasis was placed on using a verified prototype framework (i.e., R^2^-PIASS framework) to act as the conversational agent, not limiting the inputs into the AI-based GPT tools but rather making it easier to assess whether the framework prompts channelled the users' interest towards the pre-determined goal of enhancing coach learning, feedback information, and eventually athlete development (12, 13). As shown in Table 1, the R^2^-PIASS framework provided contextually relevant input themes for analyzing the conversational exchanges between semi-professional rugby union referees (representing sport coaches) and on-field players in the sport of Rugby Union.

**Table 1:** R^2^-PIASS generative pretrained transformer framework prompts.

GPT Theme	Prompt Descriptor	Prompt Specifiers
Responsiveness	Responding to the cognitive, social and health needs of players.	Acknowledging on run
		Solving problems off-ball
		Responding player needs
Game Organisation and Behaviour Management	Preventing/redirecting negative player behaviour and communication that ensures good running of the game.	Immediate command
		Preventative communication
		Redirecting negative behaviour
Proficiency and Reading the Game	Ability to organise game with minimal interruptions and for handling on-field dynamic decisions.	Running game smoothly
		Rapport/respect with players
		Directing play on the run
Communication	Method/s of communication used by the referee for referee-player interactions.	One-way communication
		Two-way communication
		Communication-play continues
		Non-verbal communication

### 3.1 Participants

There was a purposive effort (see 44) to recruit participants who were actively refereeing Rugby Union at the semi-professional level. Referees at this level of experience were known to acknowledge the importance of communicating effectively and to be able to deal with the constant accountability of performing their role (19). It is also well known that sport referees communicate more frequently than sport coaches during games, using their voices to make calls, explain decisions, and give clear instructions to players (20). Thus, sport referees were seen as a suitable metaphor for this study as they provide a substantial volume of sport-based conversational data over one game. Study participants therefore needed to be experienced in overseeing the sport of rugby union (over 6+ years active refereeing) and operating in the state-wide Division 1 Competition. Five referees provided written consent for their games to be video recorded using an Insta 360-degree camera. Based on wanting to explore whether AI-based GPT tools can be used to tag, configure, and analyze audio data for conversational performance assessments and the small number of study participants, limited personal information was collected about each referee to reduce the likelihood of a referee's identity being revealed during the research and publication processes.

### 3.2 Data collection

Data collection involved storing an audio and visual recording of each referee's game captured from a single head-mounted Insta 360-degree video camera. This recording was made continuously from the pre-match locker room, after the match referee warmed up, until the referee returned to the locker room at half time, when the camera was exchanged for another identical camera by the lead researcher to ensure sufficient battery life until the end of the match. Five Division 1 games, at five different venues, were recorded using this procedure. Unlike previous research using 360-degree video [see (21)], the Insta 360-degree camera automatically stitches images and audio together without the need to join or synchronize two 180-degree video shots together. This automated process made it possible to perform 360-degree visual scans in all directions from a single camera source to examine both the visual and audio data, including isolating the conversational dialogue occurring between the referee and players on-field.

### 3.3 Data analysis

The primary goal of this study was to explore whether freely available AI-based GPT tools can be used to tag, configure, and analyze audio data for the purpose of completing a conversational-based performance assessment. To analyze the data, we chose to employ an AI augmented thematic analysis process based on the R^2^-PIASS framework prompts [see (12, 22)]. Prompts, in AI augmented analyses, have much in common with the identification of specific features or aspects of the data that seem relevant to the research question and research context (23). Thus, rather than using an open-inductive process, patterns and relationships within the conversational data were identified using a pre-determined coding framework for the GPT prompts, purposely designed to support the analysis of rugby referee-player dialogue. Moreover, while the conversational nature of freely available AI-based GPT tools makes them an accessible interface that requires no special training or skills, the construction of precise prompting terms is known to improve the accuracy of data analysis, including the return of contextually relevant information more efficiently (12, 24).

One referee dataset was selected for analysis by means of two different freely available AI-based GPT tools: ChatGPT v4 and DeepSeek v3. ChatGPT is one of the most popular AI-based GPT sources available, having significantly more users, website traffic and overall brand recognition, making it an ideal “freely available” model for analysing the referee-player dialogue (25). DeepSeek was chosen for its ability to generate fast information with customized outputs, which can potentially improve the accuracy of answers related to LLM analyses (25). Once the GPT prompts tag, configure and analyze the audio data, the automated output results were compared with a manual content analysis of the same referee audio dataset using the same R^2^-PIASS prompts. Notably, the importance of dataset selection in LLM research cannot be overstated (26). While statistical analysis places importance on large-scale datasets at the centre of model design, development and evaluation, the enormous scale of such datasets has often been widely mythologized as beneficial to the perceived generality of trained systems (26, 27). In this project, dataset selection was therefore seen as a key issue that required careful consideration to ensure that the output parameters were robust and directly applicable to coach learning and athlete development applications.

**Figure 1:**
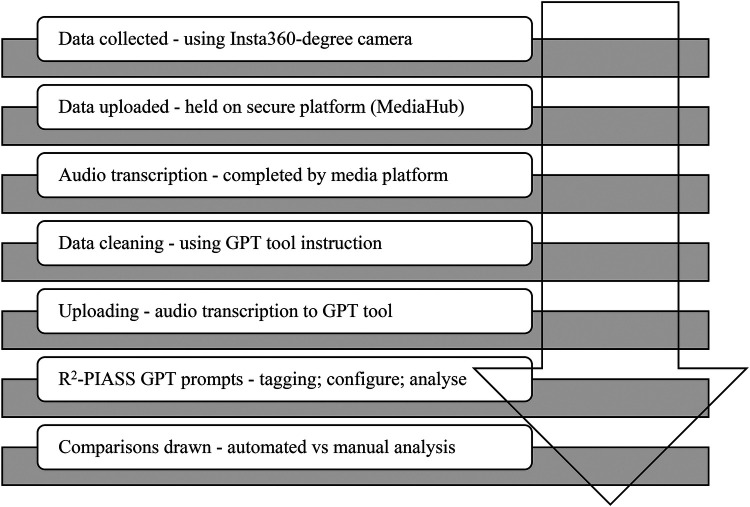
Data analysis flowchart.

Recent work by Schlangen (28), noted that datasets that map directly to a use case (e.g., automatic transcription of audio data) are suitable benchmarks “if the task is well-aligned with its real-world use case and the dataset is sufficiently representative of the data the systems would encounter in production” (p. 2). Moreover, recent work in NLP has revealed how single baseline datasets can be robust for LLM research and that models trained on incomplete inputs still perform quite well (26, 27). Considering this and based on concerns that data uploaded to freely available AI-based GPT tools is available in the public domain, this project purposively selected one audio dataset for conversational analysis. Rather than adopting the common practice of “if it is available to us, we ingest it” [see (29)], as is typical in other AI-driven, data-centric processing disciplines, we carefully selected a sufficiently representative dataset for analysis. In other words, rather than focusing on the statistical properties of datasets as a site for trying to address and mitigate bias or fabricated/hallucinated data, we intentionally chose to mitigate the impacts of noise in the input data as our simple language task is aimed at framing an input space (i.e., R^2^-PIASS prompts) and an output or action space (i.e., comparing manual and GPT tools) for an applied outcome (28). We further highlight that human judgment on natural language reasoning tasks is variable and our GPT evaluations of this task (i.e., ChatGPT v4 and DeepSeek v3) should reflect both this variability and risks of reusing data outcomes out of context (27, 28). Figure 1 outlines the procedural steps involved in the project's data analysis.

## 4 Results

The results of the study are organised into two parts. The first section compares the results of an unedited referee conversational extract captured from the Insta 360-degree video camera with an AI-cleaned data extract sourced from the same video extract using ChatGPT v4 and DeepSeek v3. The second section compares the frequency of prompt occurrences between the R^2^-PIASS manual content analysis and automated ChatGPT v4 and DeepSeek v3 R^2^-PIASS content analyses.

### 4.1 Conversational transcript comparisons

**Figure 2:**
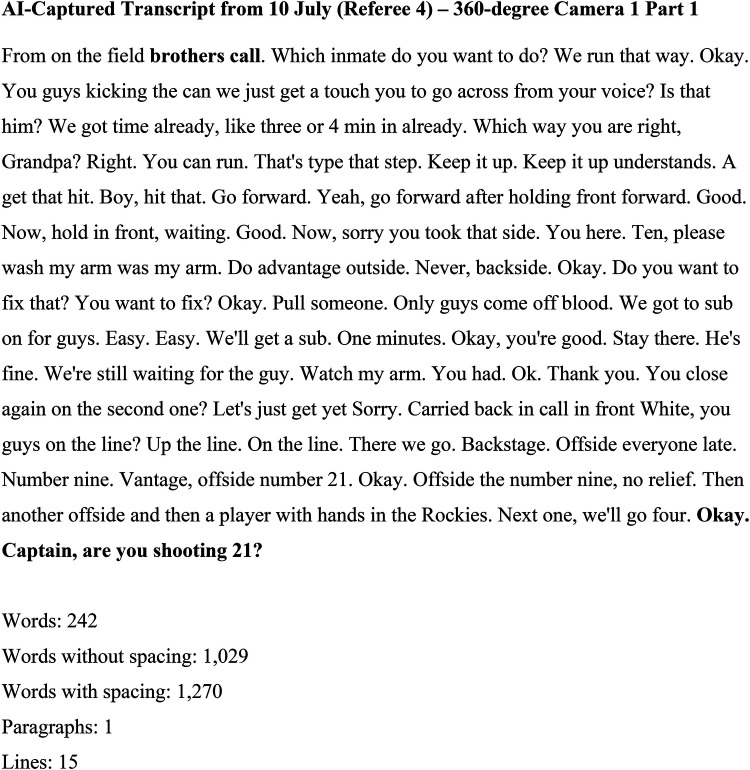
Mediahub audio transcription—source 360-degree camera*.*

Figure 2 displays an extract of Referee 4's unedited audio captured automatically from the Insta 360-degree video camera for the 10 July game. The storage platform, MediaHub, a secure video platform for university staff that avoids the risks of placing content on public distribution platforms, uses Automatic Speech Recognition (ASR) technology to instantly generate a printable transcript by converting spoken words into text. The MediaHub extract provided illustrates how the raw referee conversational data required extensive cleaning by either manual or automated process prior to uploading into a GPT tool for analysis.

**Figure 3:**
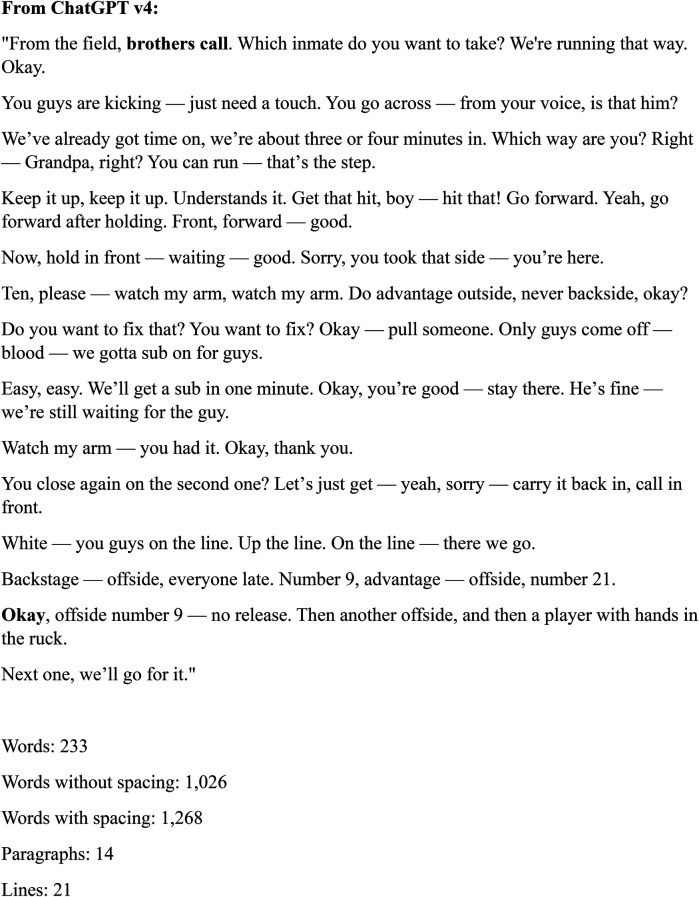
ChatGPT v4 cleaned audio transcription—source MediaHub ASR.

**Figure 4:**
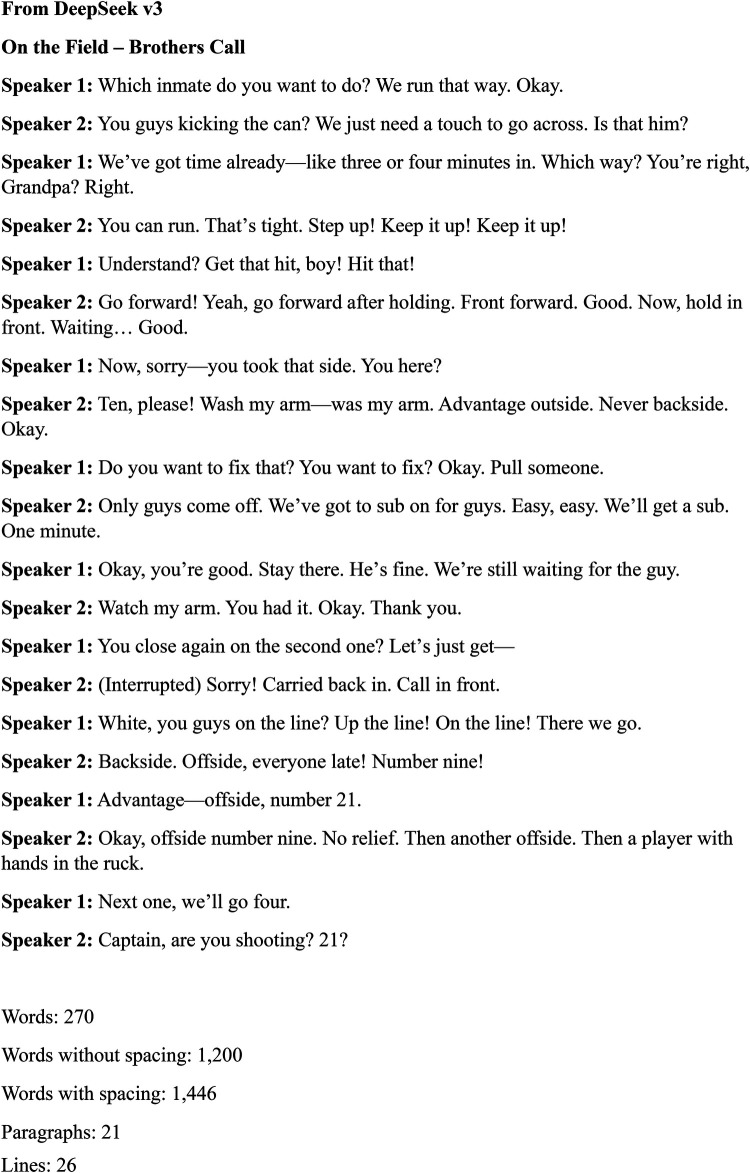
Deepseek v3 cleaned audio transcription—source MediaHub ASR.

Figures 3, 4 represent “cleaned” referee-player transcripts sourced from the same audio extract. To clean the MediaHub transcription, we entered the command “Clean this transcription:” into both freely available AI-based GPT tools followed by the full text of the unclean transcription (30). The final cleaned transcripts were reviewed only by AI. For comparison, the Review feature in word was used to calculate the number of words, words without spacing, words with spacing, number of paragraphs and number of lines in both GPT “cleaned” transcription extracts.

### 4.2 Analyzed audio data sourced using the R^2^-PIASS GPT prompts

Well considered prompts can generate GPT sourced summaries to a specified level of detail (12). For this project, numerical comparisons were drawn between a manual content analysis and ChatGPT v4 and DeepSeek v3 R^2^-PIASS content analyses of one referee dataset. Four GPT sentence prompts were designed to capture data about the R^2^-PIASS themes of Responsiveness; Game organisation and Behaviour Management; Proficiency and Reading the Game; and Communication. Sentence prompts were purposively kept simple and designed to closely align with the prompt specifiers outlined in the R^2^-PIASS framework. Table 2 outlines the sentence prompts used. While the cleaned source material could be reformatted into two separate transcripts to reflect each half of a game, in this project, the material was uploaded as one word file. Once uploaded, coding immediately began.

**Table 2:** GPT prompt specifier commands*.*

Theme	GPT Instruction/Prompt Specifier
Responsiveness	identify and group the number of times the referee responded to the cognitive, social and health needs of the players in transcript
Game Organisation	identify and group the number of times the referee prevented or redirected negative player behavior with immediate command, preventative communication, redirected negative behavior in transcript
Prof. Reading Game	identify and group the number of times the referee displayed rapport and respect, directed play on the run, ran the game smoothly in transcript
Communication	identify and group the number of times the referee used one-way communication, two-way communication, non-verbal communication and play-on communication in transcript

Table 3 displays comparisons between the frequency and percentage of responses from the ChatGPT v4 and DeepSeek v3 R^2^-PIASS content analysis and manual R^2^-PIASS content analysis. Frequency represents the number of times a specific R^2^-PIASS framework prompt specifier appeared in the audio transcript. For example, there were 116 instances of two-way communication between the referee and player/s on field manually identified, compared with 18+ instances by ChatGPT and 23 instances by DeepSeek. The average time for each coding prompt to be completed in both ChatGPT v4 and DeepSeek v3 was 2:00 min. However, while ChatGPT v4 immediately accepted and analyzed each R^2^-PIASS prompt specifier command, DeepSeek v3 required several “regenerate” commands, stating that the “server was busy; please try again later”. DeepSeek v3 also added the “approximate” symbol to the R^2^-PIASS Responsiveness results, while ChatGPT v4 added the “plus” sign to the R^2^-PIASS communication results.

**Table 3:** Comparison—frequency of GPT prompt*s.*

Theme/Prompt	ChatGPT v4		DeepSeek v3		Manual Analysis	
	*ƒ*	%	*ƒ*	%	*ƒ*	%
Responsiveness	54		∼133		72	
Acknowledging	23	42.6	∼105	78.9	57	79.1
Solve Problems	19	35.2	∼17	12.8	12	16.7
Responding	12	22.2	∼11	8.3	3	4.2
Game Organisation	53		27		102	
Immediate Command	25	47.2	12	44.4	46	45.1
Preventative Comm.	14	26.4	7	25.9	30	29.4
Redirecting Behav.	14	26.4	8	29.6	26	25.5
Prof. Reading Game	74		81		163	
Smooth Game	23	31.1	29	35.8	33	20.2
Rapport Respect	22	29.7	14	17.3	2	1.2
Directing Play	29	39.2	38	46.9	128	78.5
Communication	83+		166		866	
One-Way	43+	51.8	112	67.5	229	26.4
Two-Way	18+	21.7	23	13.9	116	13.4
Play-on/Continues	16+	19.3	9	5.4	347	40.1
Non-Verbal	6+	7.2	22	13.3	174	20.1

ChatGPT v4 and DeepSeek v3 both categorised referee-player dialogue examples and provided a “summary of instances” table at the end of each prompt command. However, DeepSeek v3 extended the process by also adding “Key Observations” and “Suggested Improvement” statements after the summary of instances table. Table 4 displays an extract of ChatGPT v4 and DeepSeek v3 response outputs for the R^2^-PIASS Responsiveness theme under the “social” prompt specifier request (i.e., identify and group the number of times the referee responded to the cognitive, social and health needs of the players in transcript).

**Table 4:** Comparison—output responses “social needs” GPT prompt*.*

R^2^-PIASS Responsiveness Theme
ChatGPT v4 Output—**Social Needs**
*ChatGPT outlined interpretation*: includes conflict management, respect, communication clarity, encouragement, or emotional acknowledgment.
**Instances: 8**
1. “Respect to the scrum already.” 2. “Sorry, you took that side — you here.” 3. “Okay, thank you.” 4. “Good luck, good luck.” 5. “I'll do my best. That's the reason he said arguments.” 6. “Captain — we're not going to have that again. If I see players instigating that, they're going to get yellows.” 7. “Hello, Jeremy — right, good.” 8. 8. “How's that looking? Good. You?”
DeepSeek v3 Output—**Social Needs**
*DeepSeek outlined interpretation:* (Referee managing player behavior, conflict, or team dynamics)
• *“You didn't even hear? Just get out. Get out! That's it.” (Conflict management)* • *“Brothers call. Which inmate do you want to do?” (Team coordination)* • *“Okay. Thank you.” (Positive reinforcement)* • *“We spoke before the game, guys—we need to get onside.” (Reminder for discipline)*
**Count:** ∼4 instances
DeepSeek v3—**Extended Responses**
**Key Observations:**
1. **Dominant Cognitive Focus**: The referee prioritizes game flow, technical rulings, and player positioning (∼80% of interactions). 2. **Minimal Social Management**: Fewer social interactions, mostly warnings or brief encouragements (∼16%). 3. 3. **Limited Health Interventions**: Rare explicit health checks, mostly tied to fouls or stoppages (∼4%).
**Suggested Improvement**: The referee could enhance player welfare by **explicitly checking on downed players** and **calming tensions more proactively** during heated moments.

## 5 Discussion

This study builds on the effectiveness of using coach-player data for practitioner learning and development (5, 31, 32). The overall goal was aimed at understanding whether a freely available AI-assisted GPT application can be a valuable resource in a sport coaches toolkit to be embedded in, replace and/or augment tasks related to coach learning, practice plans and performance feedback extracted from coach-athlete conversations. The R^2^-PIASS prompt-based conversational agent provided the framework for signposting the conversational performance parameters using two freely available AI-assisted GPT applications: ChatGPT v4 and DeepSeek v3. The concept behind using freely available AI-assisted GPT applications was based on their accessibility; that is, most sporting clubs in Australia operate on a volunteer basis where coaches wanting to upskill or learn more about athlete development need to invest a significant amount of their own time and energy to upskilling processes, potentially impacting how and when they can learn (33). Freely available AI-assisted GPT applications that offer the latest GPT technologies were therefore seen as being within reach, allowing coaches to search for sport information and upload files for both athlete- and self-reflection analyses. Thus, with a focus on leveraging readily available AI-based tools for advancing coach learning and athlete development, the discussion section provides an overview of the project's results that broadly follows Braun and Clarke's six-phase thematic analysis framework [see (23, 34)], including (1) familiarization with data, (2) GPT data and data cleaning, (3) generating and refining GPT prompt specifier commands, (4) generating responses, and (5) implications for using GPT platforms for coach learning, practice design, and performance analyses.

### 5.1 Familiarization with data

Of relevance to the approach used in this study, the lead author had extensive experience in sport as a coach educator and national sport coach. The second author worked in the field gaining prolonged engagement with the data in order to support meaning making, evidence and argument related to referee-player dialogue statements (35). Therefore, while GPT platforms can support the rapid identification of key content [see (36, 37)], familiarization with the project data using ChatGPT v4 and DeepSeek v3 platforms played a minor role in this project, as reliance on them was not needed for gaining a deep understanding of the context of coach-athlete conversations which underpinned the rest of this project's analytical approach.

### 5.2 GPT data and data cleaning

Conversational analysis in this project required cleanly captured on-field conversational data. The Insta 360-degree video camera provided a suitable platform for recording coach-player dialogue statements as the camera was mounted on the head of the sport referee while they conducted a game. This meant that previse audio data was recorded in preparation for performance analysis. However, current methods for generating automated transcriptions from audio data are still not well advanced (38). Indeed, similar to previous studies on the benefits of using automated transcription platforms [e.g. (30, 36, 37),], this study found that even advanced computational models for audio transcription were not as accurate as traditional transcription methods as they lacked understandings of context, accents, rugby-specific terminologies related to rulings, and non-verbal cues (22, 39). Moreover, while ChatGPT v4 and DeepSeek v3 platforms offered a dynamic data cleaning approach, the quality of the original transcription input from MediHub meant that an authentic coach-player transcription was not fully achieved. Moving forward, we argue that the LLM would need to be trained to answer the information needed in a similar context for which the dataset was originally created to try and ensure that the dataset authentically addresses the input needs of coach learners who work in specialized practical settings (37).

### 5.3 Generating and refining GPT prompt specifier commands

The main finding related to generating and refining GPT prompt commands was that both ChatGPT v4 and DeepSeek v3 could provide sport coaches with a basic analysis of coach-player conversational interactions. Notably, this aligns with how earlier studies uncovered the ways in which LLM tools can act as consultants and statistical advisors when wanting to find answers and solutions for analysis topics (40). However, the results of this project also highlight that the generation of GPT prompts comes with caveats and limitations, including whether the prompt commands provide sufficient context and instructional detail for the GPT tool to search and report on what is required. For example, using one overarching sentence as the search parameter for analysing conversational dialogue in this project stresses that conversational data may have been not identified due to the tool not understanding what was needed. Moreover, overlaps in communicative-based search terms such as preventative communication, one-way communication, two-way communication, and non-verbal communication may have mislead the LLM, potentially lowering output accuracy. This confirms what both Khraisha et al. (41) and Pawlick (45) recently illustrated; that is, the importance of prompt engineering and lack of a universally optimal prompt template for all GPT models. For sport coaches wanting to use GPT platforms, this implies that it would be beneficial to create a detailed plan for prompt engineering in advance of the performance analysis processing stage.

### 5.4 Generating output responses

Since GPT platforms such as ChatGPT v4 and DeepSeek v3 rely on sophisticated LLMs, they also require considerable computational power to generate responses from the source prompts received (13). This means that character limits are applied to the output responses regardless of whether someone is using the freely available or subscription GPT model. This limitation shaped the prompt commands used in this project, meaning that our single prompt did not contain a lot of background information to ensure we achieved the maximum character numbers when generating the output responses. Overall, both ChatGPT v4 and DeepSeek v3 provided detailed analyses for each of the four input prompts. However, when compared with the manual content analysis results, DeepSeek v3 generated more human-like responses of the referee transcript material compared to ChatGPT v4 in the Responsiveness category and addition of “Key Observations” and “Suggested Improvement” statements. Singh et al. (42) noted a similar outcome when comparing ChatGPT and DeepSeek models, highlighting that the latest DeepSeek model was designed with optimized efficiency, bias reductions, and provision of more customized responses. For sport coaches, this suggests that DeepSeek might provide a more effective analysis tool than other GPT models in terms of reasoning and non-reasoning capabilities, such as self-verification, reflection, and long conversations (42).

### 5.5 Implications for coach learning, practice designs, performance analyses

This study represents one of the initial efforts to analyze a coach-athlete conversational dataset using freely available GPT tools and a pre-determined, context-specific, prompt-based framework (i.e., R^2^-PIASS). Overall, the results illustrated that ChatGPT v4 and DeepSeek v3 models could extract quantitative and qualitative information from the source material using simple prompt specifiers. Implications for how coaches can use this technology to support their own learning, practice designs, and performance analyses (e.g., analysis of training sessions or post-match interviews) were the efficiencies both platforms provided in relation to cost, usability, accessibility and convenience. For example, both platforms were freely available and had similar input character limits. Both platforms were available 24/7 and while DeepSeek did display some issues in this project related to connectivity, further prompting resulted in the output material ultimately being generated. The results also illustrated that sport coaches can design input prompts themselves or ask the GPT model to generate input prompts for them. In line with Dizon (22), sport coaches can also conveniently dial up or down the content material being searched to get something into a comprehensible range, clean or transform coaching data to make it easier for the platform to understand what is required, and upload a range of source material such as coach-player conversations captured over a season for longitudinal performance-analyses. However, the primary benefit of using freely available GPT tools such as ChatGPT v4 and DeepSeek v3 as search platforms were the time savings produced by the automated analysis. For example, the manual content analysis of the coach-player conversational data took 7 h and 20 min, whereas the time for the same prompt specifiers in ChatGPT v4 and DeepSeek v3 took an average of 2 min.

Despite the strengths of using freely available GPT tools in dialogue analysis, it is imperative to also recognise the associated risks and pitfalls for coaching learning, practice designs and performance analysis processes. Indeed, our results for generating the analysis outcomes using the four theme categories were consistent with previous studies that illustrated how the simplistic research design is limited in its generalisability (40). Better data cleaning practices (i.e., overcoming automated transcription issues that may lower AI-driven data analyses), and a triangulation of the analysis outputs with sport coaches might also contribute to better understandings of whether GPT tools hold promise for transforming sport coaching practices and coach learning (13, 40). Issues with GPT tools understanding rugby-specific terms and referee phrases were also encountered. The one referee dataset also limited the strength and robustness of the applicable statistical outcomes and qualitative GPT dialogue feedback. Furthermore, similar to Hwang et al. (43), this study did not distinguish between hallucinated and unreported outcome items. For example, LLMs may propagate factual inaccuracies when trained on datasets containing hallucinated data, misinformation or fabricated content (27). Even though drawing such distinctions could have been insightful, previous studies have noted that in some GPT-based analysis tasks, such as prompt generation, users can struggle to articulate their needs to LLMs, thereby requiring users to not simply accept the LLM's output but rather taking extensive time to initially verify the correctness of the recommendations produced (37, 43). Finally, the tensions between keeping the input data within the context and ensuring that the context does not breach privacy issues must be acknowledged, as all data uploaded to freely available GPT platforms is in the public domain (12).

## 6 Conclusion

Pinpointing patterns in conversational datasets can offer sport coaches valuable insights into their own coaching strengths and weaknesses and provide information that supports their own learning, practice designs, and performance analyses. This study represents one of the initial efforts at analysing a coach-athlete conversational dataset using freely available GPT tools and a pre-determined, context-specific, prompt-based framework (i.e., R^2^-PIASS). Overall, the study found that freely available GPT platforms can offer sport coaches an efficient way of analyzing conversational data. By quickly setting the scene with discipline-specific information, sport coaches can carefully craft GPT-prompts that seek specific outputs from conversational datasets. At the same time, GPT platforms need to be trained with contextually appropriate input data to conclusively answer questions about domains that use specialized terminologies and rules. Moreover, there are risks involved when coaches rely on inaccurate AI feedback for athlete development decisions. Further investigations that engage GPT platforms for coach-athlete dialogue analysis are therefore required to ascertain the true relevance and potential of using this type of technology for coach learning and athlete development. Put simply, while freely available GPT platforms offer tremendous potential for supporting sport coaches with cutting-edge insights into conversational-based performance assessments, the reality is that crafting well-designed prompts to obtain reliable, accurate and contextually relevant responses from automatically transcribed data requires further development.

## Data Availability

The data analyzed in this study is subject to the following licenses/restrictions: Dataset belongs to the Queensland University of Technology. Requests to access these datasets should be directed to katherine.obrien@qut.edu.au.
